# Distinct metabolic patterns during microglial remodeling by oleate and palmitate

**DOI:** 10.1042/BSR20190072

**Published:** 2019-04-05

**Authors:** Bruno Chausse, Pamela A. Kakimoto, Camille C. Caldeira-da-Silva, Adriano B. Chaves-Filho, Marcos Y. Yoshinaga, Railmara Pereira da Silva, Sayuri Miyamoto, Alicia J. Kowaltowski

**Affiliations:** 1 Departamento de Bioquímica, Instituto de Química, Universidade de São Paulo, São Paulo, SP, Brazil; 2 Institute of Physiology and Pathophysiology, University of Heidelberg, D-69120 Heidelberg, Germany

**Keywords:** fatty acid, inflammation, immunometabolim, microglia, metabolic reprogramming, mitochondrial dysfunction

## Abstract

Microglial activation by oleate and palmitate differentially modulates brain inflammatory status. However, the metabolic reprogramming supporting these reactive phenotypes remains unknown. Employing real-time metabolic measurements and lipidomic analysis, we show that both fatty acids promote microglial oxidative metabolism, while lipopolysaccharide (LPS) enhances glycolytic rates. Interestingly, oleate treatment was followed by enrichment in storage lipids bound to polyunsaturated fatty acids (PUFA), in parallel with protection against oxidative imbalance. Palmitate, in turn, induced a distinct lipid distribution defined by PUFA linked to membrane phospholipids, which are more susceptible to lipid peroxidation and inflammatory signaling cascades. This distribution was mirrored by LPS treatment, which led to a strong pro-inflammatory phenotype in microglia. Thus, although both oleate and palmitate preserve mitochondrial function, a contrasting lipid distribution supports differences in fatty acid-induced neuroinflammation. These data reinforce the concept that reactive microglial profiles are achieved by stimulus-evoked remodeling in cell metabolism.

## Introduction

Microglia are resident macrophages of the central nervous system (CNS), presenting multiple roles on the maintenance of brain homeostasis [1]. These cells are phenotypically flexible and control several process in CNS development and physiology, such as synaptic maturation and plasticity [2]. Once exposed to pathological signals, microglia are activated to more reactive states that are supported by defined molecular and morphological remodeling [3]. Although microglial activation is protective under many conditions, it has also been related to uncontrolled neuroinflammation, a process involved in several brain disorders [1,2].

Mechanisms controlling different microglial reactive states are not yet fully understood. An emerging concept in immunology is that distinct metabolic programming occurs depending on cellular inflammatory status [4]. Indeed, pro-inflammatory microglia and macrophages induced by lipopolysaccharide (LPS) show marked suppression in mitochondrial respiration in parallel to enhanced glycolytic rates [5,6]. On the other hand, anti-inflammatory activation such as that induced by interleukin-4 (IL-4) is more flexible and can be sustained by different metabolic pathways [7,8]. Although microglial metabolism in overt activation has been detailed, the metabolic reprogramming supporting intermediate microglial reactive states is not known.

Dietary fatty acids are important modulators of brain inflammation [9]. Palmitate, a saturated fatty acid, has been suggested to be a central trigger of diet-induced hypothalamic inflammation, a process which leads to loss of appetite control and obesity [10,11]. Notably, saturated fatty acids affect hypothalamic inflammation directly through microglial activation [12,13]. The monounsaturated fatty acid oleate, in turn, has been shown to alleviate inflammation and prevent obesity development [14]. Indeed, an *in vitro* study suggests that oleate is able to directly inhibit LPS-induced microglial activation [15]. Although the effects of oleate and palmitate on neuroinflammation have been intensely investigated, the metabolic reprogramming sustaining fatty acid-induced microglial reactive states remains unknown.

Here, we employed real time metabolic measurements and lipidomic analysis to uncover the metabolic reprogramming that sustains microglial remodeling by fatty acids. Our data suggest that microglia exposed to oleate and palmitate are supported by oxidative metabolism. In addition, distinct lipid composition and distribution indicate differences in fatty acid-induced inflammatory tonus.

## Methods

### Cell cultures

**Figure 1:**
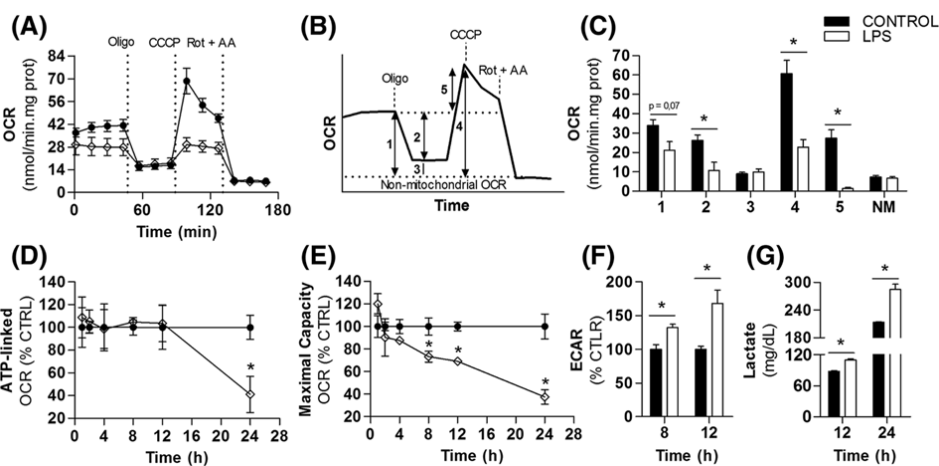
Glycolysis is up-regulated before changes in mitochondrial ATP production during microglial activation BV2 cells were treated with 100 ng/ml LPS followed by real time metabolic measurements. (**A**) Real time OCR recorded after 24 h incubation with LPS. Baseline respiration was modulated by sequential additions of 1 µg/ml Oligo, 2 µM CCCP and 1 µM Rot plus 1 µg/ml AA. (**B**,**C**) Representation of mitochondrial respiratory states calculated from data shown in panel A. Numbers in panel B correspond to the following OCRs represented in panel C: (1) mitochondrial baseline respiration, (2) ATP synthesis-linked respiration, (3) proton leak-driven respiration, (4) maximal mitochondrial respiratory capacity, (5) spare respiratory capacity, and (NM) non-mitochondrial respiration. (**D**) Microglial ATP-linked respiration and (**E**) maximal respiratory capacity after 1, 2, 4, 8, 12, and 24 h LPS. (**F**) Basal extracellular acidification rate and (**G**) extracellular lactate accumulation. Values represent averages ± SEM from two (**G**) or three (**A**–**F**) independent experiments with three to five replicates for each condition and were compared using *t* tests. Data in panels D–F represent relative changes between LPS and control groups at the same time point. ^*^P<0.05 vs control. Abbreviations: AA, antimycin A; CCCP, carbonyl cyanide-p-trifluoromethoxy-phenylhydrazone; OCR, oxygen consumption rate; Oligo, oligomycin; Rot, rotenone.

BV2 microglial cells were cultured in DMEM medium (25 mM glucose, 1 mM pyruvic acid, and 2 mM glutamine) supplemented with 100 IU/ml penicillin/streptomycin and 10% fetal bovine serum (FBS) at 37°C and 5% CO_2_. For experiments, cells were seeded in plates at concentrations described for each method. After 24 h, the culture medium was replaced by treatment medium (high glucose DMEM without FBS). For experiments displayed in Figure 1, a 5 mg/ml LPS was directly diluted in treatment medium to a final concentration of 100 ng/ml. Control groups were incubated with LPS-free treatment medium. When experiments included exposure to lipids, cells were incubated with fatty acids and LPS in the presence of 0.25% (37.8 µM) fatty acid-free BSA. Fatty acid/BSA complexes were freshly prepared before experiments, following recommendations highlighted in Alsabeeh et al. [16]. Briefly, for fatty acid stock solutions, oleate and palmitate were dissolved in water to produce a 10 mM solution and heated to 65°C until the solution was totally clear. BSA was dissolved in water to produce a 10% (m/v) solution at 37°C. Fatty acid/BSA conjugation was performed by adding 500 µl of lipid stock solution to 2.5 ml of BSA stock solution every 5 min at 37°C. After four fatty acid additions, the volume was completed with 500 µl of water to achieve a final concentration of 4 mM fatty acid/5% BSA. Solutions were then filtered (0.22 µm) and diluted 20-fold before incubation with the cells. Conjugation produced a fatty acid:BSA ratio of approximately 5:1. BSA stock solutions were also filtered (0.22 µm), diluted 40-fold and used as vehicle (control groups) and to dilute LPS to 1 µg/ml. Cell viability was assessed by Trypan Blue exclusion method performed in a TC20™ Automated Cell Counter (BioRad).

### Real-time metabolic analysis

Cellular oxygen consumption rates (OCR) and extracellular acidification rates (ECAR) were measured using a XF24 Analyzer (Seahorse Technology Agilent) as described by Cerqueira et al. [17]. Briefly, 20000 cells/well were seeded on Seahorse cell culture plates using a total volume of 600 µl of supplemented culture medium. After 24 h, cells were incubated with treatment solutions (treatments are described in the figure legends). Before respiratory measurements, cells were washed and incubated for 1 h with 500 µl respiratory medium (25 mM glucose DMEM plus 1 mM pyruvic acid and 2 mM glutamine; sodium bicarbonate and FBS were absent) in atmospheric air at 37°C. After equipment calibration, baseline respiration measurements were followed by 1 µg/ml oligomycin addition to determine ATP-linked and proton leak-driven respiration. CCCP (carbonyl cyanide-p-trifluoromethoxy-phenylhydrazone, 2 µM), a mitochondrial uncoupler, was added to induce maximal respiratory capacity. Non-mitochondrial respiration was determined after the addition of 1 µM rotenone, a complex I inhibitor, plus 1 mg/ml antimycin A, a complex III inhibitor. All respiratory modulators were used at ideal doses established by preliminary titrations, as instructed by the manufacturer. ECAR values shown correspond to the fourth baseline respiration measurement, just before oligomycin addition. OCR and ECAR values were normalized to protein content, measured using the Bradford method.

### Lactate and NO^.^ detection

Lactate content in the supernatants was determined using commercial kits, following the manufacturer’s instructions (Labtest, Brazil). NO**^.^** release was estimated by quantifying supernatant nitrite content, a product of NO**^.^** oxidation, using the Griess reaction. Briefly, 100 µl of treated cell supernatant was incubated with 50 µl solution A (100 µM sulfanilamide + 0.05 N HCl) and 50 µl solution B (350 µM N-1-napthylethylenediamine dihydrochloride [NED]) for 10 min. The absorbance was then read at 540 nm. Standard nitrite curves were used for data calibration.

### Western blots

Cell lysates were diluted in Laemmli buffer and proteins were separated using a 12% polyacrylamide denaturating gel. Proteins were transferred to nitrocellulose membranes and incubated with 1:1000 anti-very long-chain acyl-CoA dehydrogenase (anti-VLCAD, ab155138, Abcam) or 1:500 anti-TOM20 (sc136211, Santa Cruz). Anti-ACTB (1:5000) (ab8226, Abcam) was used as a loading control. Fluorescent secondary antibodies (IRDye® anti-mouse and anti-Rabbit, 1:20000) were added to the membranes and bands were obtained using a near-infrared Odissey system. Bands were semi-quantified by densitometric analysis using ImageJ software.

### Lipidomic analysis

Prior to lipid extraction, a mixture of internal standards was added to the samples to allow for lipid class semi-quantification (Supplementary Table S1). Lipid extraction was performed following the method established by Matyash [18]. In summary, 10^6^ cells were resuspended in 10 mM phosphate buffer containing 100 µM deferoxamine mesylate followed by cold methanol, internal standards, and methyl-tert-butyl-ether (MTBE) addition. After 1 h stirring at room temperature, water was added and samples were kept over ice for 10 min. After centrifugation at 10000 g for 10 min at 4°C, the supernatant containing the total lipid extracts (TLE) was transferred to a new tube and dried under N_2_ gas. Dried TLE were dissolved in isopropanol (100 µl) and the injection volume was set at 1 µl. TLE were analyzed by electrospray ionization time-of-flight mass spectrometry (ESI–TOF MS, Triple TOF 6600, Sciex, Concord, U.S.A.) interfaced with ultra-HPLC (UHPLC Nexera, Shimadzu, Kyoto, Japan). Spectra were obtained in positive-ion and negative-ion mode and analyzed using Analyst® 1.7.1. MS/MS data were analyzed using PeakView® and lipid species were identified by visual prospection based on compound mass and fragments using Excel macros and Lipid Maps (http://www.lipidmaps.org). Lipid quantification was obtained using MultiQuant®, where precursor ion peak areas were normalized to internal standards.

### Reduced/oxidized glutathione quantification

Reduced (GSH) and oxidized (GSSG) glutathione were quantified using a liquid chromatography system coupled to mass spectrometry. Briefly, after treatments, 5 × 10^6^ cells per condition were lysed in 180 μl of iced PBS with 18 µl of extraction buffer (20% trichloroacetic acid and 10 mM diethylenetriaminepentaacetic acid) plus 2 µl of internal standard (200 µg/ml n-acetyl cysteine; NAC). This mixture was vortexed for 1 min followed by 15 min incubation over ice. Sample pH was adjusted to 2.0 by mixing with 200 µl of mobile phase A (0.75 mM ammonium formate, 0.01% formic acid, 1% methanol) and cellular debris were subsequently pelleted by centrifugation at 5000 × *g* for 10 min at 4°C. Supernatants were collected and analyzed by ESI–TOF MS (Triple TOF 6600, Sciex, Concord, U.S.A.) operated in the positive mode interfaced with a UHPLC (Nexera, Shimadzu, Kyoto, Japan). The chromatographic method was developed using a Kinetex C18 analytical column (100 mm × 2.10 mm, 2.6 µm) (Phenomenex, Torrance, CA, U.S.A.) eluted with a mobile phase of 0.75 mM ammonium formate/0.01% formic acid/1% methanol (solvent A) and methanol (solvent B) at 0.2 ml/min. Elution was performed as follows: 1% solvent B held for 5 min followed by a gradient step to 80% from 5 to 6 min, held at 80% B for 4 min and restored to 1% from 10 to 11 min. Finally, the column was equilibrated until 20 min of the run. The column temperature was set at 25°C and injection volume was 10 μl. Data acquisition was performed using Analyst® 1.7.1 with an ion spray voltage of 5.5 kV and the cone voltage at 80 V. The curtain gas was set at 25 psi, nebulizer and heater gases at 45 psi and interface heater of 450°C. The collision energies used for each compound were 22 eV for GSH; 32 eV for GSSG; 25 eV for NAC. The areas obtained from extracted-ion chromatogram of specific fragments were used for quantification of GSH (*m/z* 308.1 → 179.0462), GSSG (*m/z* 613.2 → 355.0741 plus *m/z* 307.1 → 177.0328), and the internal standard (NAC) (*m/z* 164.0 → 76.0215) using Multiquant® software. GSH and GSSG concentrations were obtained plotting the GSH/NAC and GSSG/NAC ratios against a standard calibration curve.

### qRT-PCR

To evaluate CD36, inducible nitric oxide synthase (iNOS), arginase 1 (ARG1), tumor necrosis factor α (TNF-α), and interleukin 1β (IL-1β) expression, cells were seeded in 6 well plates (2 × 10^5^ cells/well) and treated as described in figure legends. Cells were then lysed using TRIzol® reactant followed by RNA extraction and cDNA synthesis (High Capacity System, Life Technologies). Real-time RT-PCR analysis of gene expression was performed using an ABI Prism 7500 sequence detection system (Life Technologies). Each PCR reaction contained 40 ng of cDNA, 200 nM of each specific TaqMan assay (Life Technologies), and ribonuclease-free water to a final volume of 20 μl. Real-time data were analyzed using Sequence Detector System 1.7 from Applied Biosystems. β-actin gene expression was used as an endogenous control.

### Cytokine quantification

Cytokine content in supernatants was analyzed using BD™ CBA Mouse Inflammation Kits (BD™) following manufacturer’s instructions. In summary, supernatants were incubated with capture beads for the detection of monocyte chemoattractant protein-1 (MCP-1) and TNF, and the mouse inflammation PE (Phycoerythrin) detection reagent for 2 h at room temperature. Wash buffer (1 ml) was then added to each assay tube followed by centrifugation at 200 ***g*** for 5 min. Supernatants were discarded and bead pellets were ressuspended in 300 µl wash buffer. PE (Phycoerythrin) fluorescence intensity of each sandwich complex revealed the concentration of that cytokine, which was calibrated using standard curves. Samples were acquired using a flow cytometer (BD FACSCanto™ platform) and analyzed using FCAP Array™ software. Cytokine levels were normalized to cell protein content in each well.

### Statistical analysis

Data were analyzed using GraphPad Prism Software. Figures represent averages ± SEM and were compared using *t* tests or ANOVA (followed by Tukey’s *post* test), as described in figure legends. Two-tailed *P-*values under 0.05 were considered significant. Statistical analysis for the lipidomics data, such as the heatmap and principal component analysis (PCA) were performed using MetaboAnalyst (http://www.metaboanalyst.ca).

## Results

### LPS up-regulates glycolysis before inducing a decline in mitochondrial ATP production

LPS-activated microglia switch from oxidative metabolism to anaerobic glycolysis for ATP production [5]. To gain insight into mitochondrial adaptations supporting this metabolic shift, BV2 cells were treated with LPS for 24 h, and analyzed by real-time oxygen consumption monitoring. LPS treatment resulted in changes in microglial mitochondrial respiration under basal conditions (Figure 1A,C), or the respiratory rates obtained in the absence of any mitochondrial modulators subtracted from non-mitochondrial respiration, as indicated by ‘1’ in explanatory Figure 1B. OCR in the presence of oligomycin, an ATP synthase inhibitor, revealed that ATP synthesis-linked OCR (number 2 in Figure 1B) was significantly diminished by LPS (Figure 1A,C). To investigate whether ATP production was limited by alterations in mitochondrial respiratory capacity, we added the uncoupler CCCP, a membrane-permeable proton carrier that promotes a collapse in the mitochondrial inner membrane potential, leading to maximal oxygen consumption. LPS-activated microglia displayed a pronounced decrease in maximal respiratory capacity (number 4 in Figure 1B, data shown in Figure 1A,C). Indeed, mitochondrial spare respiration, the additional respiratory capacity recruited when cells are energetically challenged (Figure 1B, number 5), was virtually absent after LPS treatment (Figure 1A,C). Rotenone plus antimycin A, inhibitors of respiratory complexes I and III, respectively, were added at the end of each experiment to determine non-mitochondrial OCR, which was similar in LPS and control groups (Figure 1A,C). Noticeably, LPS-induced suppression in mitochondrial function was not related to decreased cell viability (Supplementary Figure S1).

To evaluate whether these changes were triggered earlier during microglial activation, mitochondrial respiration was also monitored 1, 2, 4, 8, and 12 h after LPS exposure. ATP-linked OCRs remained unaltered over this period (Figure 1D), although maximal respiratory capacity was progressively decreased after 8 h (Figure 1E).

Glycolysis up-regulation is an important step in some immune responses [19]. Increases in glycolytic rates have been shown to enable inflammatory intermediate synthesis, replacing oxidative phosphorylation as the main cellular ATP source. To assess adaptations in glycolytic flow, we measured ECAR and extracellular lactate accumulation in a time-dependent manner. Interestingly, we detected an increase in both the ECAR (Figure 1F) and extracellular lactate (Figure 1G) before the attenuation in ATP-linked respiration, at 8 and 12 h, respectively. This suggests the glycolytic rate is enhanced before mitochondrial ATP production is impaired in LPS-activated microglia, and is therefore not a consequence of limited respiration.

### Fatty acids maintain microglial oxidative phosphorylation

**Figure 2:**
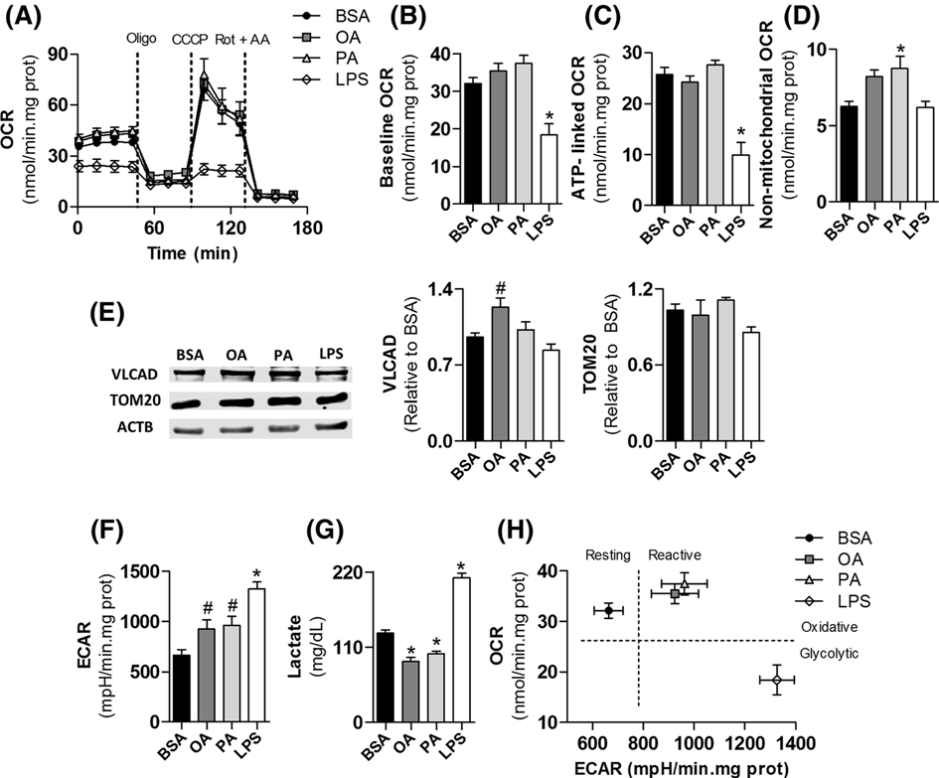
Microglial activation by palmitate and oleate is sustained by oxidative metabolism, while LPS activation is glycolytic BV2 cells were treated for 24 h with 0.25% (37.8 µM) BSA, 200 µM oleate, 200 µM palmitate or 1 µg/ml LPS followed by real time metabolic measurements. (**A**) Real time OCR. Baseline respiration was modulated by sequential additions of 1 µg/ml Oligo, 2 µM CCCP, and 1 µM Rot plus 1 µg/ml AA. (**B**) Microglial mitochondrial baseline respiration, (**C**) ATP-linked respiration, and (**D**) non-mitochondrial oxygen consumption. (**E**) Representative bands and quantification by densitometry of SDS–PAGE Western blots of BV2 cells lysates in different treatments, (**F**) basal ECAR, and (**G**) extracellular lactate accumulation. (**H**) Microglial metabolic phenotyping. Baseline OCR and ECAR values were used to depict metabolic states. Values represent averages ± SEM from four (**A**–**F**) or five (**G**) independent experiments and were compared using one-way ANOVA followed by Tukey’s *post* test. ^*^P<0.05 vs BSA. ^#^*P*<0.05 vs LPS. Abbreviations: AA, antimycin A; OA, oleate; Oligo, oligomycin; PA, palmitate; Rot, rotenone.

The metabolic reprograming sustaining fatty acid-induced microglial reactive states remains unknown, although their effects on neuroinflammation have long been investigated [9]. To assess how metabolism is affected during microglial remodeling by fatty acids, BV2 cells were treated with 200 µM palmitate or oleate for 24 h followed by metabolic profiling. LPS was used as positive control and BSA as a vehicle for the fatty acids [16]. While LPS promoted a marked decrease in mitochondrial respiration, fatty acid-treated microglia presented unchanged mitochondrial function (Figure 2A). Palmitate treatment increased baseline respiration by approximately 16% in all experiments. However, the average baseline OCR was not statistically different from the control BSA group (Figure 2B). Indeed, mitochondrial respiration linked to ATP synthesis was not changed by fatty acids (Figure 2C). Although both fatty acids increased non-mitochondrial respiratory rates, this effect was significant only in the palmitate-treated group (Figure 2D). Levels of TOM20, an outer mitochondrial membrane protein that is a marker for mitochondrial mass, were similar between all treatments, suggesting LPS-induced suppression in respiratory rates is not related to changes in mitochondrial content (Figure 2E).

We next evaluated ECARs and lactate accumulation to assess whether microglial glycolytic flow is modulated by fatty acid treatment. Both oleate and palmitate induced a slight increase in ECAR that was significantly lower than that induced by LPS (Figure 2F). Notably, microglial lactate release was decreased after fatty acid treatment, suggesting increments in ECAR are not linked to glucose fermentation (Figure 2G). To assess if the enhancement in ECAR was associated to altered lipid oxidation machinery, we measured the content of the VLCAD, a β-oxidation enzyme. Oleate induced an increment in VLCAD content that was significantly different from the LPS-treated group (Figure 2E).

To further uncover the metabolic phenotype induced by fatty acid treatment, basal OCR was plotted against basal ECAR for all groups (Figure 2G). This representation is a useful tool to depict metabolic states. A comparative increase along the axes indicates a more oxidative or glycolytic profile and is also related to enhanced metabolic rates. All treatments induced active metabolic phenotypes, but LPS-activated microglia became glycolytic while both fatty acids induced an oxidative metabolic programming. Overall, these data indicate that microglial remodeling by fatty acids does not involve the loss in OCR clearly observed in LPS-activated cells (Figure 2G).

Arginine metabolism is also an important regulator of innate immune responses [20]. In pro-inflammatory profiles, arginine is preferentially employed for NO**^.^** production through iNOS. In anti-inflammatory cells, arginine oxidation through the urea cycle is stimulated by higher ARG1 expression [5]. Unlike LPS, we found that fatty acid treatment did not change iNOS expression or NO**^.^** production (Table 1). Conversely, ARG1 expression was higher in oleate-treated cells compared with LPS-treated microglia (Table 1). Indeed, inflammatory cytokines were strongly induced by LPS, while fatty acids did not modulate their production either at the transcriptional or translational level (Table 1).

**Table 1:** Production of inflammatory mediators

	BSA	Oleate	Palmitate	LPS
*mRNA* (relative to BSA)				
**TNF-α** (*n*=5)	1.020 ± 0,10	1.232 ± 0.14	1.150 ± 0.17	72.25 ± 4.85 *
**IL-1β** (*n*=5)	1.040 ± 0.14	0.828 ± 0.11	1.324 ± 0.33	826.8 ± 141.3 *
**iNOS** (*n*=5)	1.006 ± 0.05	1.552 ± 0.64	1.883 ± 0.69	16297 ± 6508 *
**ARG1** (*n*=5)	1.182 ± 0.37	1.584 ± 0.39 #	0.952 ± 0.22	0.053 ± 0.01
*Protein secretion* (ng/ml.mg prot)				
**TNF-α** (*n*=3)	0.131 ± 0.05	0.144 ± 0.04	0.069 ± 0.01	12.27 ± 0.91 *
**MCP-1** (*n*=3)	6.010 ± 1.89	4.932 ± 1.19	5.376 ± 0.72	14.41 ± 2.59 *
*Nitrite accumulation* (μM)				
**NO_2_** (*n*=5)	1.055 ± 0.10	1.322 ± 0.10	0.702 ± 0.11	17.16 ± 2.13 *

Values are means ± SEM and were compared using one-way ANOVA followed by Tukey’s *post* test. ^*^ *P*<0.05 vs BSA. ^#^*P*<0.05 vs LPS.

### Microglial lipid profile is distinctively remodeled by oleate, palmitate, and LPS

**Figure 3:**
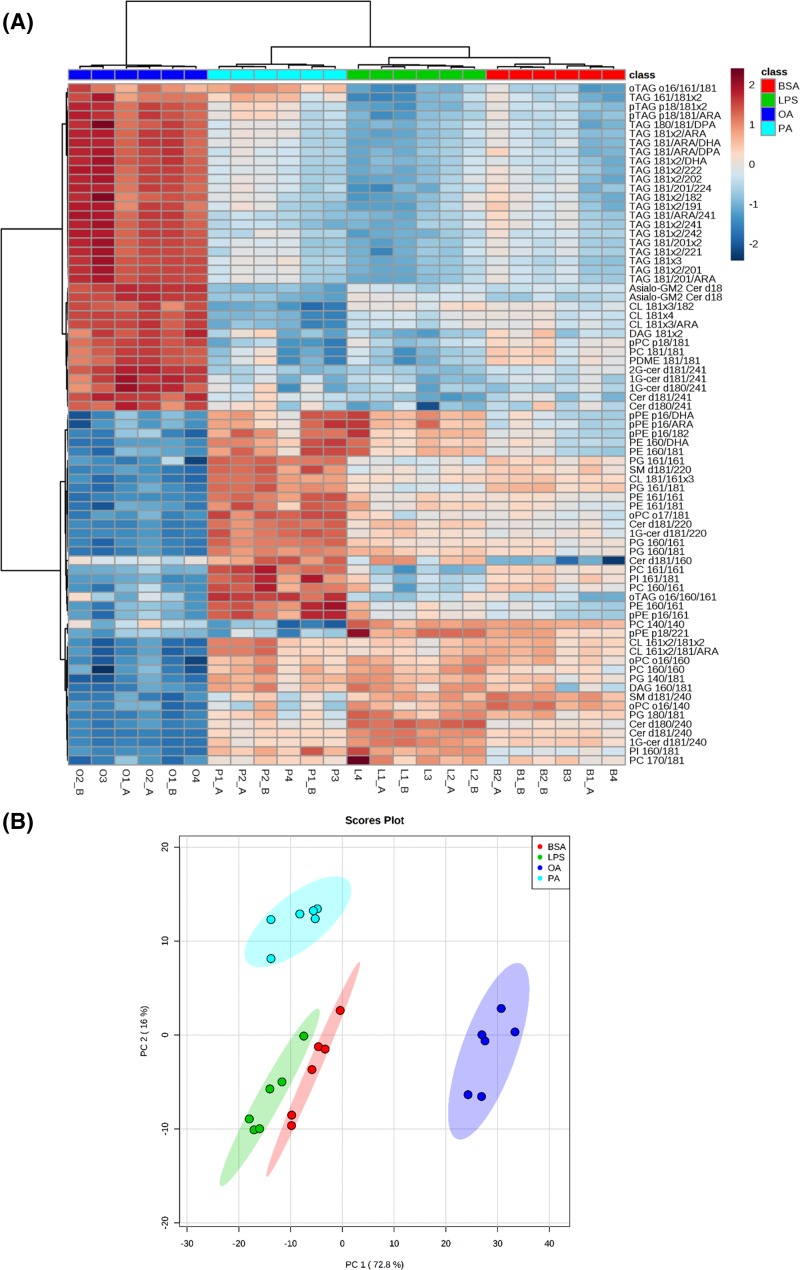
Microglial lipid profile is distinctly remodeled by oleate, palmitate, and LPS treatment BV2 cells were treated for 24 h with 0.25% (37.8 µM) BSA, 200 µM oleate, 200 µM palmitate, or 1 µg/ml LPS followed by lipid extraction and lipidomic analysis. (**A**) Heat-map presenting top 75 altered lipids (according to their *P*-values). Each row corresponds to a lipid where the detection level in each sample was normalized by the average detection in all samples. The complete data of significantly altered lipid molecular species is displayed in Supplementary Table S2. (**B**) PCA. Total log-normalized lipid concentrations used to depict the scatter plots of first and the second principal components (PC1 and PC2, respectively). Abbreviations: OA, oleate; PA, palmitate.

Lipids can alter inflammatory responses modulating cell signaling, energy homeostasis, and/or redox balance [14]. Studies show that alternative lipid composition, oxidation, and storage patterns are related to specific immune profiles [21–24]. We performed an untargeted lipidomic analysis to gain further insights into how fatty acid and LPS treatments affect microglial metabolic reprogramming. Lipidomic analysis revealed that oleate, palmitate, and LPS induce distinct cellular lipid profiles (Figure 3). Indeed, 172 out of 176 monitored lipids were significantly altered between treatments (*P*<0.05, Supplementary Table S2). A heat-map depicting the top 75 altered lipids shows that major species enriched by palmitate and LPS were markedly decreased by oleate and that triglycerides were by far the most enriched lipids in oleate treatment (Figure 3A). Moreover, PCA efficiently separated four groups in the first two principal components (PC1 and PC2, explaining 88.8% of the data variance). These data collectively suggest that oleate, palmitate, and LPS generate unique lipid profiles in reactive microglia relative to each other and compared with BSA (Figure 3B). A complete list of detected lipids as well as how their content was modulated in the experiments is presented in Supplementary Table S2.

**Figure 4:**
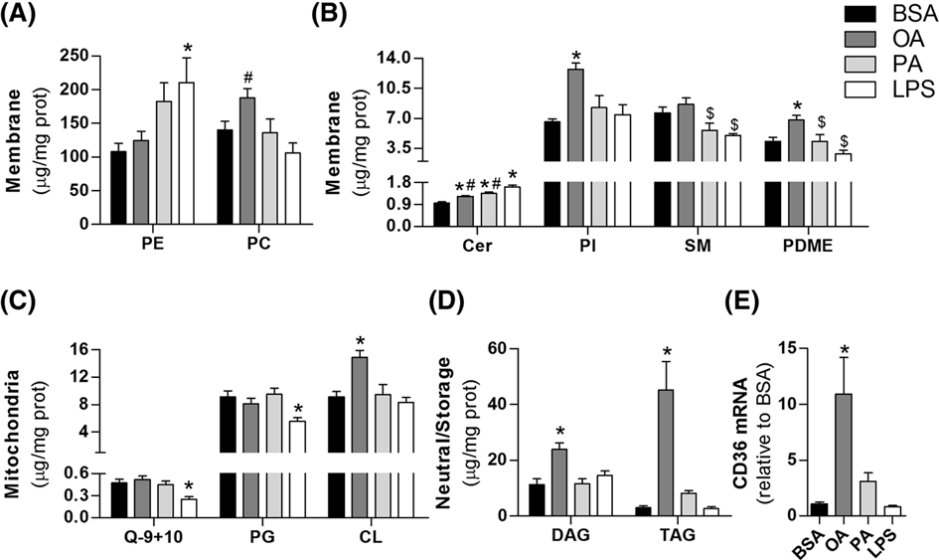
Major lipid classes are modulated during microglial reprogramming BV2 cells were treated for 24 h with 0.25% (37.8 µM) BSA, 200 µM oleate, 200 µM palmitate or 1 µg/ml LPS followed by lipid extraction and lipidomic analysis. (**A**–**D**) Microglial lipidome represented by 11 lipid classes detected by lipidomic analysis. (**E**) CD36 gene expression. Values represent averages ± SEM from five (**E**) or six (**A**–**D**) independent experiments and were compared using one-way ANOVA followed by Tukey’s *post* test. *P<0.05 vs BSA. ^#^*P*<0.05 vs LPS. ^$^*P*<0.05 vs OA. Q-9+10 values cannot be taken as absolute concentrations, see Supplementary Table S1 for CoQ quantification. Abbreviations: Cer, ceramide; CL, cardiolipin; PC, phosphatidylcholine; PDME, phosphatidyldimethylethanolamine; PE, phosphatidylethanolamine; PG, phosphatidylglycerol; PI, phosphatidylinositol; Q-9+10, Coenzymes Q9 + Q10; SM, sphingomyelin.

Microglial lipidome details are presented in Figure 4. Fatty acid and LPS treatment strongly affected major classes of membrane (Figure 4A,B), mitochondrial (Figure 4C), and neutral/storage lipids (Figure 4D). Notably, LPS promoted a reduction in coenzyme Q9 and Q10 levels (Figure 4C) that paralleled decreases in mitochondrial respiratory capacity (Figures 1C and 2A). Ceramide content was boosted by all treatments, although oleate and palmitate induced lower levels than LPS (Figure 4B). The most distinct lipid profile was induced by oleate. In two different clustering approaches, the oleate-linked lipidome is clearly separated from other treatments (Figure 3A,B). Indeed, different lipid classes such as cardiolipin, phosphatidylinositol, and phosphatidylcholine were exclusively up-regulated by oleate (Figure 4A-C). Distinctively, oleate induced a marked increase in triacylglycerol (TAG) and diacylglycerol (DAG) contents, suggesting increased storage lipid synthesis (Figure 4D). These changes were followed by a pronounced increment in CD36 expression, which has been described as an important regulator of immune responses (Figure 4E).

### Oleate induces protective fatty acid distribution in reactive microglia

**Figure 5:**
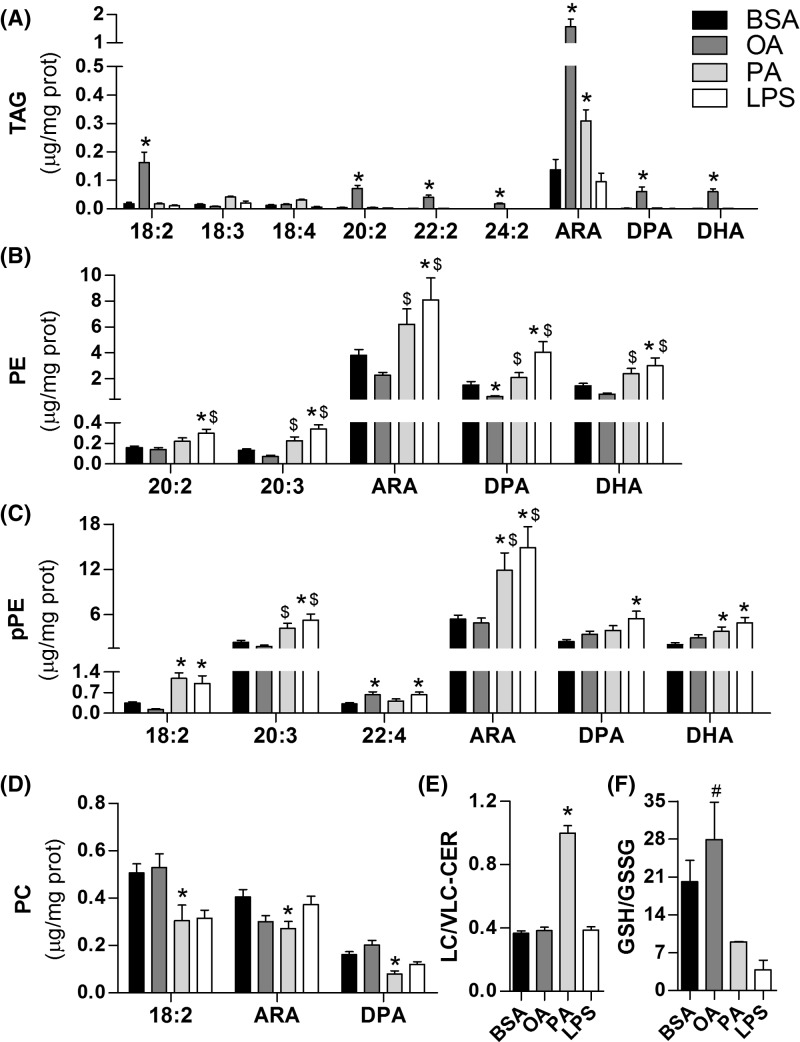
Fatty acid distribution in major lipid classes reveals striking differences between oleate versus palmitate and LPS treatments BV2 cells were treated for 24 h with 0.25% (37.8 µM) BSA, 200 µM oleate, 200 µM palmitate or 1 µg/ml LPS followed by lipidomic analysis. PUFA incorporation into (**A**) TAG, (**B**) PE, (**C**) pPE, and (**D**) PC. (**E**) Ratio of long chain and very long chain fatty acid-enriched ceramides. (**F**) GSH/GSSG ratio. Values represent averages ± SEM from two (**F**) or six (**A**–**E**) independent experiments and were compared using one-way ANOVA followed by Tukey’s *post* test. * *P*<0.05 vs BSA. ^#^*P*<0.05 vs LPS. ^$^*P*<0.05 vs OA. Abbreviations: ARA, arachidonic acid; DHA, docosahexaenoic acid; DPA, docosapentaenoic acid; OA, oleate; PA, palmitate; PC, phosphatidylcholine; PE, phosphatidylethanolamine; pPE, plasmalogens of phosphatidylethanolamine.

Lipidomic analysis also demonstrated that the composition of fatty acids linked to TAG and phospholipids differed broadly between groups. Notably, polyunsaturated fatty acids (PUFA) were enriched in most TAGs from oleate-treated cells (Figure 5A, Supplementary Table S2). Conversely, palmitate and LPS induced an increment in phospholipids bound to PUFA (Figure 5B–D). Palmitate also enhanced long n-acyl chain ceramides compared with very long n-acyl chain ceramides (up to 22 carbons and more than 22 carbons, respectively, Figure 5E), a feature often related to pro-inflammatory responses [25]. Saturated and unsaturated fatty acid incorporation into TAG and phospholipids was also distinct between groups (Supplementary Figure S2).

Fatty acids located in membranes and inside lipid droplets present different susceptibility to lipid oxidation [26]. To test whether the divergent lipid distribution was related to alterations in oxidative balance, we measured the ratio of GSH to GSSG in cells treated with LPS or fatty acids. This ratio is often used as a measurement of oxidative imbalance since GSH is converted to its oxidized form in the presence of oxidants. Interestingly, the GSH/GSSG ratio was higher in oleate than in palmitate and LPS treatments (Figure 5F), suggesting microglia exposed to oleate are protected against oxidative imbalance.

## Discussion

Microglial activation has often been studied as a bimodal process, with focus on the interplay between extreme pro-inflammatory or anti-inflammatory profiles such as those induced by LPS and IL-4, respectively [1]. Based on similarity to these patterns, intermediate reactive profiles have been ascribed as M1 (pro-inflammatory) or M2 (anti-inflammatory), although they do not always correspond to the full effects of LPS or IL-4. Indeed, recent debates have established that, upon different stimuli, microglia and macrophages can assume a wide range of reactive phenotypes, varying in activation mechanism and inflammatory tonus [1,2,27,28]. Thus, we hypothesized that the metabolic programming supporting microglial reactive states could also change according to the stimulus applied. Confirming this notion, we show that LPS, palmitate, and oleate induce microglial reactive phenotypes that are sustained by distinct remodeling in metabolism and lipid homeostasis.

The BV2 cell line has often been used to study microglial biology *in vitro* because it maintains many microglial features [29]. Although it is a myc-transformed lineage, these cells present responses in cell metabolism [5,30,31], mitochondrial morphology [32,33], and redox balance [33,34] similar to those shown by inflamed primary microglia and macrophages. Here, upon LPS treatment, these cells presented marked production of pro-inflammatory factors (Table 1) in parallel with enhancement in glycolysis rates (Figure 1), as seen in previous studies [5,35,36]. Of note, our temporal analysis added a new finding to this process: increments in glycolysis and reduction in mitochondrial maximal respiratory capacity precede the limitation in mitochondrial ATP production (Figure 1 D–F). These results suggest that the LPS-induced metabolic switch is not triggered by impairments in mitochondrial ATP synthesis. Instead, it may be regulated in parallel with the production of inflammatory intermediates affecting mitochondrial respiratory machinery. Different processes have been related to mitochondrial functional restructuring during innate immune cell activation. These include respiratory complex IV inhibition by NO**^.^** ([5,31], Table 1), reduction in coenzyme Q content (Figure 4C), alterations in mitochondrial morphology [33,38], decreased isocitrate dehydrogenase expression [20,39] and the recently described metabolite itaconate, which competitively inhibits respiratory complex II [40–42]. Nevertheless, the contribution of each metabolic parameter as well how they synergistically trigger changes in mitochondrial function remain to be determined.

Palmitate is considered the main effector of diet-induced neuroinflammation [9]. Indeed, high fat diet consumption is followed by an enrichment in palmitate content in the hypothalamus, in parallel with microgliosis and pro-inflammatory cytokine release [11,12]. Microglial depletion experiments have further suggested that hypothalamic inflammation is dictated by palmitate-induced reactive microglia [12,13]. Due to its pro-inflammatory character, microglial activation by palmitate has been directly compared with LPS-elicited phenotypes. However, our results suggest that these profiles are controlled by different metabolic reprogramming (Figure 2).

We show that, unlike LPS, palmitate-treated microglia are sustained by oxidative metabolism (Figure 2). Indeed, metabolic phenotypes depicted in Figure 2H suggest that oleate and palmitate-treated microglia are sustained by oxidative metabolism and present increased metabolic rates compared with the control group. This is in line with recent studies on macrophages demonstrating that palmitate treatment induces an oxidative phenotype sustained by lipid oxidation [43]. The inhibition of this pathway exacerbates inflammatory tonus, suggesting intact mitochondrial function might alleviate palmitate-related inflammation [22,43]. Thus, lipid oxidation can represent a central metabolic module in fatty acid-induced reactive microglia. Here, we detected a slight increase in VLCAD content in oleate-treated microglia and an enhancement in ECAR in parallel with a decrease in lactate release in both palmitate and oleate treatments (Figure 2). ECARs are well known to be altered by other pathways modulating extracellular pH such as the Krebs cycle and do not always reflect direct modulation of glycolytic flow [44], further suggesting fatty acids can induce lipid oxidation in microglia. Interestingly, non-mitochondrial respiration was increased by palmitate (Figure 2D), an effect potentially related to enhanced peroxisomal β-oxidation and/or oxidant production. The increase in plasmalogen content in palmitate-treated microglia (Figure 5) further suggests peroxisomal activation, although there is, to our knowledge, no direct evidence in the literature associating plasmalogen biosynthesis with peroxisomal β-oxidation [45]. In addition to differences in metabolism, an interesting study showed that macrophages isolated from obese patients and treated with palmitate *in vitro* present a completely different cell surface protein signature from that observed in macrophages isolated from patients with cystic fibrosis or activated with LPS *in vitro* [46]. These results emphasize that palmitate and LPS promote mechanistically distinct pro-inflammatory phenotypes in microglia and macrophages.

Although palmitate has been considered a direct inducer of inflammation in microglia and macrophages, we were not able to detect changes in inflammatory mediator contents, either at the transcriptional or translational level (Table 1). This may be a consequence of alterations in the lipid overload model employed by different studies. Indeed, distinct inflammatory profiles have been described with changes in lipid concentration and availability (which is usually determined by fatty acid/BSA proportion) in cell culture lipotoxicity models [16]. Palmitate-induced inflammation is often verified when higher concentrations (>400 μM) or higher fatty acid/BSA ratios (>5:1) are used [43,47]. Conversely, lower concentrations (<200 μM) as well lower fatty acid/BSA proportions (>3:1) have even been shown to prevent pro-inflammatory microglial activation [48–50]. Here, we used a lipotoxicity protocol similar to that used in studies employing hypothalamic slice cultures which have supported the central role for microglia in palmitate-induced hypothalamic inflammation ([palmitate] = 200 μM; fatty acid:BSA = 5:1) [12,13]. This suggests that, in addition to the moderate lipotoxicity protocol, inflammatory intermediate production in our study can be limited by the lack of secondary signals controlling microglial activation by palmitate *in situ*. It is worth noting, however, that the lipid profiles of palmitate and LPS-treated cells were relatively similar in respect to high PUFA content linked to major phospholipids (Figure 5). Additionally, arachidonic acid concentration in phosphatidylethanolamine and plasmalogens of phosphatidylethanolamine, two of the most abundant lipid classes (Figures 4A and 5B–C), were higher than both BSA and oleate treatments. The high availability of PUFA at the membrane level and their high susceptibility to oxidation may lead to increased production of lipid mediators, such as the oxidized products of arachidonic acid, putatively known as pro-inflammatory eicosanoids [51].

In contrast to palmitate, oleate is considered protective against diet-induced inflammation [14]. Rodents fed with oleate-rich diets present a global decrease in metabolic inflammation. *In vitro* studies suggest that oleate also promotes an anti-inflammatory effect by directly inhibiting LPS-induced microglial activation and avoiding lipid peroxidation [15,52]. Our results show that, although microglia treated with both palmitate and oleate are supported by oxidative metabolism (Figure 2), lipid composition and distribution was broadly distinct in oleate versus palmitate and LPS treatments (Figure 3). Oleate-treated cells were enriched in TAG concomitantly with higher CD36 expression (Figure 4D,E). In a recent study, Rohwedder et al. [53] showed that oleate directly induces fatty acid storage and lipid droplet formation by activating the long-chain fatty acid receptor FF4. The contribution of CD36 to this process remains unclear, although its putative function is the preferential uptake of long-chain fatty acids [54,55]. In addition, the role of CD36 in inflammatory signaling is controversial. There is evidence for a direct participation of CD36 in phospholipase A2 activation, which is related to the release of PUFA linked to phospholipids, prostaglandin E2 synthesis, and metabolic inflammation [56]. In contrast, Huang et al. [23] demonstrated that macrophage anti-inflammatory activation is dependent on CD36-mediated lysosomal acid lipase activation. Of note, CD36 expression is controlled by nuclear receptors, such as PPAR-γ, which have been recognized as central nodes in anti-inflammatory responses and can also be activated by fatty acids [57,58].

Although the role of CD36 in oleate-treated cells is uncertain, our results revealed a significant decrease in PUFA linked to phospholipids compared with palmitate and LPS treatments (Figure 5B,C). More specifically, the significant decrease in PUFA linked to phospholipids corresponded to a quantitative increment of PUFA within TAG in oleate-treated microglia (Figure 5A). The preferential location of PUFA within lipid droplets rather than membrane phospholipids can affect inflammatory tonus in two manners: (I) preventing the synthesis of inflammatory lipid mediators and (II) avoiding propagation of lipid peroxidation at the membrane level. In fact, LPS-activated microglia present an increase in eicosanoid content along with an increase in cytosolic phospholipases co-localized with lipid droplets [59,60]. In contrast, oleate promotes a decrease in phospholipases associated with lipid droplets, leading to protection against arachidonic acid release [60]. This mechanism may explain the oleate-induced enrichment in PUFA within lipid droplets, which in turn may prevent lipid peroxidation at the membrane level and avoid oxidative stress-mediated inflammatory signaling. This is in line with recent evidence suggesting that lipid droplets, in addition to functioning as energy storage units, can act as antioxidant organelles, shielding PUFA chains from peroxidation [26]. The higher GSH/GSSG ratio further supports the hypothesis that oleate-induced lipid distribution and oxidative balance generates a protective phenotype in microglia (Figure 5F).

Although our results add important and new insights into the mechanisms controlling microglial remodeling by fatty acids, the use of an immortalized cell lineage does bring some limitations. Recent studies have demonstrated that microglia insertion in the brain parenchyma is crucial for the maintenance of microglial homeostatic features [61,62]. Moreover, signal integration in the brain microenvironment can widely modulate microglial responses. Adaptive immune cell infiltration as well as neuronal and astrocytic signals can either protect or worsen lipid-induced neuroinflammation [9]. Therefore, additional studies must be performed to understand how alterations in microglial metabolism and lipid homeostasis affect brain inflammatory status *in situ*. Overall, our data suggest that although microglia activated by oleate or palmitate are energetically supported by oxidative metabolism, a distinct lipid distribution might determine their divergent inflammatory profiles. Indeed, palmitate-induced lipid composition changes were similar to those promoted by LPS, which in turn, was associated with strong pro-inflammatory activation.

## Supporting information

**Supplementary Figure S1:** 

**Supplementary Figure S2:** 

**Supplemental Table S1:** 

## Abbreviations

ARG1: arginase 1
ATP: adenosine triphosphate
CCCP: carbonyl cyanide-p-trifluoromethoxy-phenylhydrazone
CNS: central nervous system
ECAR: extracellular acidification rate
ESI: electrospray ionization
FBS: fetal bovine serum
GSH: reduced glutathione
GSSG: oxidized glutathione
IL-1β: interleukin 1β
IL-4: interleukin-4
iNOS: inducible nitric oxide synthase
LPS: lipopolysaccharide
MCP-1: monocyte chemoattractant protein-1
NAC: n-acetyl cysteine
NO: nitric oxide
OCR: oxygen consumption rate
PCA: principal component analysis
PUFA: polyunsaturated fatty acid
TAG: triacylglycerol
TLE: total lipid extract
TNF-α: tumor necrosis factor α
U-HPLC: ultra-HPLC
VLCAD: very long-chain acyl-CoA dehydrogenase
