# Supplementary figures and images for: Distinct metabolic patterns during microglial remodeling by oleate and palmitate
Source: Biosci Rep. 2019 Apr 5;39(4):BSR20190072. doi: 10.1042/BSR20190072 (PMC6449521; doi:10.1042/BSR20190072)

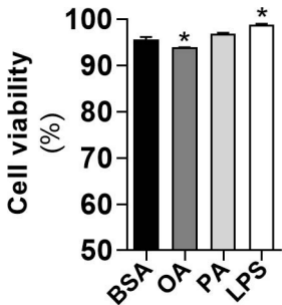

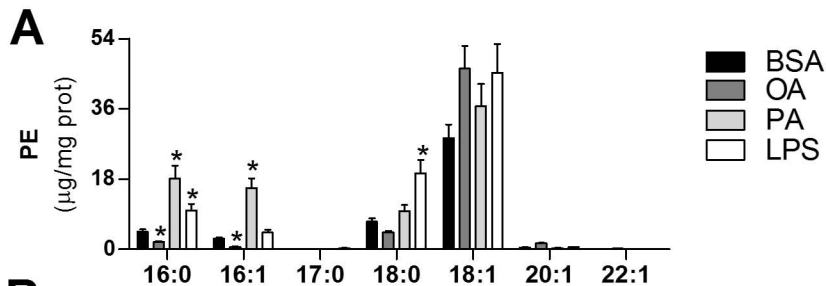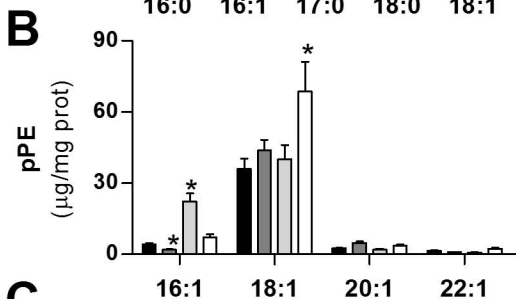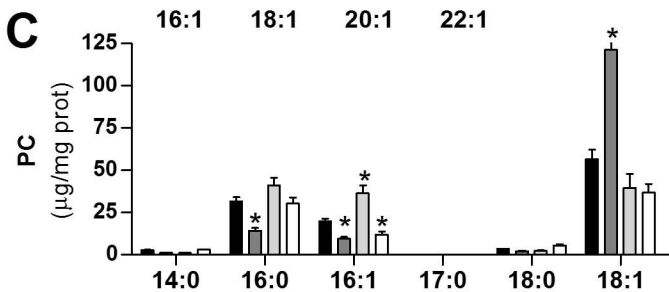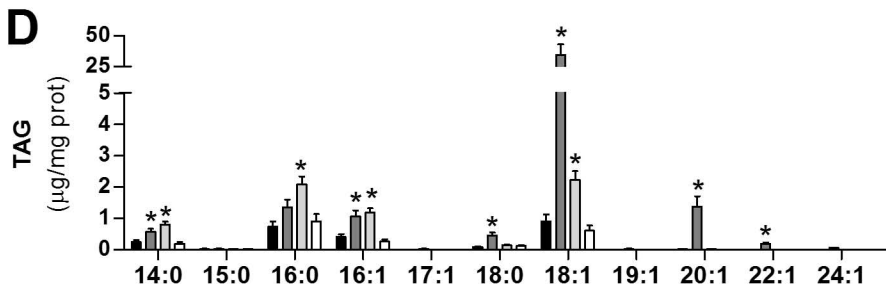

Supplement: Supplementary file 1 [file bsr-39-bsr20190072_Supp1.pdf]
